# COVID-19 Associated Ischemic Stroke and Hemorrhagic Stroke: Incidence, Potential Pathological Mechanism, and Management

**DOI:** 10.3389/fneur.2020.571996

**Published:** 2020-10-27

**Authors:** Zilan Wang, Yanbo Yang, Xiaolong Liang, Bixi Gao, Meirong Liu, Wen Li, Zhouqing Chen, Zhong Wang

**Affiliations:** ^1^Department of Neurosurgery and Brain and Nerve Research Laboratory, The First Affiliated Hospital of Soochow University, Suzhou, China; ^2^Department of Orthopedics, The First Affiliated Hospital of Soochow University, Suzhou, China; ^3^Department of Neurology, The First Affiliated Hospital of Soochow University, Suzhou, China; ^4^Department of Neurology, The Second Affiliated Hospital of Soochow University, Suzhou, China

**Keywords:** COVID-19, SARS-CoV-2, ischemic stroke, hemorrhagic stroke, nervous system

## Abstract

The outbreak of the novel coronavirus infectious disease 2019 (COVID-19) caused by the SARS-CoV-2 virus has rapidly spread around the world. Increasing evidence has suggested that patients with COVID-19 may present neurological symptoms, and cerebrovascular diseases are one of the most frequent comorbidities. The markedly elevated D-dimer levels in patients with acute ischemic stroke suggests that SARS-CoV-2 infection may induce an inflammatory response and trigger a hypercoagulation state, thus leading to acute ischemic stroke. Cardioembolism and atherosclerosis in patients with COVID-19 infection may also increase the risk of ischemic stroke. The reduction of the angiotensin-converting enzyme II (ACE2) caused by SARS-CoV-2 binding to the ACE2 receptor can lead to abnormally elevated blood pressure and increase the risk of hemorrhagic stroke. Additionally, the cytokine storm induced by the immune response against the viral infection increases the risk of acute stroke. The management for COVID-19 patients with stroke is not only based on the traditional guidelines, but also based on the experience and new instructions from healthcare workers worldwide who are combatting COVID-19.

## Introduction

As COVID-19 has rapidly spread worldwide, increasing evidence suggests that SARS-CoV-2 may also invade the central nervous system and induce neurological symptoms (1–3). Research from Wuhan reported neurologic manifestations in 36.4% of 214 COVID-19 patients (4). An increasing number of studies have revealed that in addition to the typical respiratory symptoms such as fever and dry cough, patients with COVID-19 may develop neurological manifestations, ranging from mild to severe (4–9).

Stroke presents as one of the most frequent causes of death and disability all around the world. More than 9,000 new stroke cases occur each day in China (10). Previous studies have suggested that cerebrovascular disease is an independent risk factor for severe cases of COVID-19 infection (11). The risk of cross-infection and lack of experienced stroke care experts during the COVID-19 pandemic have impacted stroke centers and caused a worldwide drop of over 30% in the number of patients with stroke or transient ischemic attacks (TIA) seeking emergency care, which could affect the prognosis in these patients (10, 12–15). What is worse, being in quarantine alone during the epidemic may increase the risk of missing the therapeutic window if the patient does not seek care in a timely way. Thus, new guidelines for the management of patients with stroke in the context of the COVID-19 pandemic are urgently needed. This review aims to summarize current evidence of the epidemiology and potential mechanisms of various cerebrovascular diseases with COVID-19 to provide clinical insight for the management of such patients.

## Possible Mechanisms Underlying the Central Nervous System Invasion

**Figure 1:**
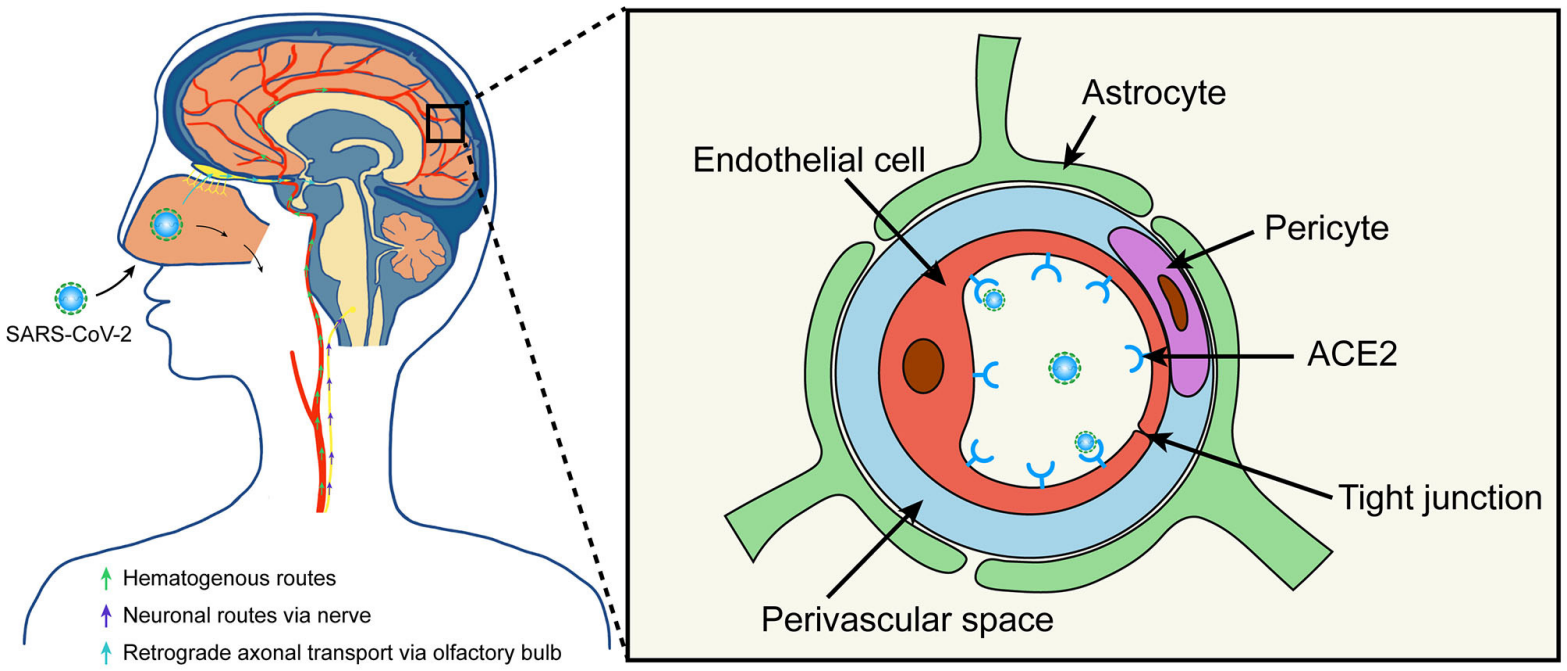
Possible routes for SARS-CoV-2 to enter the brain. SARS-CoV-2 may enter the central nervous system through hematogenous or neuronal routes. The virus may enter the brain *via* cerebral circulation after systemic circulatory dissemination. Moreover, the virus may enter the brain *via* central or peripheral nerve, especially the retrograde axonal transport from the olfactory bulb. Additionally, SARS-CoV-2 can bind and engage with the ACE2 receptor in the capillary endothelium to damage the blood-brain barrier. ACE2, angiotensin-converting enzyme II.

As with other neurotropic respiratory viruses, two major pathways, the hematogenous and neuronal retrograde, have been proposed as possible routes for SARS-CoV-2 to enter the central nervous system (CNS) (16–18) (Figure 1) After systemic circulatory dissemination following infection of the lung, the virus may enter the brain *via* cerebral circulation (17). According to a postmortem examination of a COVID-19 patient, viral-like particles in the brain capillary endothelium were observed actively budding across endothelial cells, suggesting the hematogenous route as the most likely pathway for SARS-CoV-2 entering the brain (19). Second, cases of olfactory dysfunction in COVID-19 patients suggests retrograde axonal transport *via* the olfactory bulb as another possible entry route (9, 20, 21).

Similar to SARS-CoV, SARS-CoV-2 exploits the angiotensin-converting enzyme II (ACE2) receptor for cell entry (22). Previous studies identified that ACE2 receptors were expressed in the brain, which suggests the potential for nervous system invasion of SARS-CoV and SARS-CoV-2 (17, 23, 24). Both SARS-CoV and SARS-CoV-2 have been detected in postmortem examinations from the brains of SARS or COVID-19 infected patients (19, 24). Given that the SARS-CoV-2 spike protein can interact with ACE2 expressed in the capillary endothelium, the virus may also damage the blood-brain barrier and enter the CNS by attacking the vascular system (17). Moreover, a case of COVID-19 with encephalitis has also been confirmed to contain SARS-CoV-2 in the cerebrospinal fluid (CSF) (6).

## COVID-19 Infection May Induce Venous and Arterial Thromboembolism

Severe COVID-19 infection can cause the release of pro-inflammatory cytokines, which induce the expression of tissue factor (TF) by endothelial and mononuclear cells and leads to coagulation activation and thrombin generation (25, 26). It has been reported that the procoagulant state caused by COVID-19 infection may induce venous and arterial thromboembolism. A retrospective analysis from Wuhan revealed that abnormal coagulation parameters, especially markedly elevated D-dimer and fibrin degradation product levels are associated with poor prognosis in patients with COVID-19 (*P* < 0.05) (27). D-dimer is a degradation product of cross-linked fibrin and is a frequently used marker of hypercoagulable and thrombotic events (28). Moderately elevated levels of D-dimer are associated with the risk of venous and arterial events in patients with vascular disease (28). A multi-center study evaluated the incidence of the composite outcome of the venous and arterial thrombotic complications (including symptomatic acute pulmonary embolism, deep-vein thrombosis, ischemic stroke, myocardial infarction, or systemic arterial embolism) in all 184 COVID-19 patients admitted to the ICU (29). The cumulative incidence of the composite outcome was 31%, of which venous thromboembolism made up 27% and arterial thrombotic events (all ischemic strokes) made up 3.7% (29). Another similar study from Italy also analyzed the venous and arterial thromboembolic complications in 388 COVID-19 patients (30). The results showed that, despite the use of anticoagulant prophylaxis, the rate of venous and arterial thromboembolic complications in hospitalized COVID-19 patients accounts for ~8% of the included patients (30). Additionally, more than half of these diagnoses of thromboembolic events were made within the first 24 h of hospital admission (30). Ischemic stroke was diagnosed in nine (2.5%) patients, and in six patients stroke was the primary reason for hospitalization. They also showed that the D-dimer levels rapidly increased in non-survivors during hospitalization (30). These clinical studies suggest the urgent need for developing pharmacological thrombosis prophylaxis strategies in severe COVID-19 patients.

## COVID-19 Infection With the New Onset of Ischemic Stroke

The reported incidence of acute ischemic stroke in COVID-19 patients ranges from 2.5 to 5%. Stroke usually develops several days after COVID-19 infection. In rare cases, it can be the primary reason for the hospitalization of COVID-19 infection (25). According to research conducted during the epidemic, large vessel occlusion was more common in COVID-19 infected patients with stroke.

The first study focused on the neurological manifestations of patients with COVID-19 from the epicenter of the pandemic in Wuhan, China, and reported neurological complications in 78 (36.4%) of 214 patients (4). Acute cerebrovascular disease was more common among patients with severe COVID-19 than those with a non-severe disease (5 [5.7%]: four patients with ischemic stroke and one with cerebral hemorrhage who died later of respiratory failure; vs. 1 [0.8%]: one patient with ischemic stroke; *P* = 0.03) (4). Of the six patients with acute cerebrovascular disease, two arrived at the emergency department presenting with sudden onset of hemiplegia but without any typical symptoms of COVID-19 (4). To note, reported patients with severe infection were found to have higher D-dimer levels than that of patients with non-severe infection (4). A retrospective study by Li et al. (31) on acute cerebrovascular disease from Wuhan, China showed that of the 219 patients with COVID-19, 10 (4.6%) developed acute ischemic stroke and one (0.5%) had a cerebral hemorrhage. The median duration from the first symptoms of COVID-19 infection to stroke was 10 days. Of the 10 patients with ischemic stroke, five were large vessel occlusion, two were small vessel occlusion, and three were of cardioembolic type according to the Trial of Org 10172 in Acute Stroke Treatment (TOAST) classification (31). Moreover, older patients (75.7 ± 10.8 vs. 52.1 ± 15.3 years) with risk factors such as hypertension, diabetes, and previous medical history of cerebrovascular disease are more likely to develop acute cerebrovascular disease (31). In addition, an increased inflammatory response and hypercoagulable state were observed in these patients as reflected in C-reaction protein [51.1 (1.3–127.9) vs. 12.1 (0.1–212.0) mg/L, *P* < 0.05] and D-dimer [6.9 (0.3–20.0) vs. 0.5 (0.1–20.0) mg/L, *P* < 0.001] (31). According to their findings, the significantly increased inflammatory response could be the cause of abnormal blood coagulation function in the early-stage and could be one of the main reasons for the new onset of cerebrovascular disease (31).

A case series (25) from the UK reported six patients with acute ischemic stroke and COVID-19. All six patients had large vessel occlusion with markedly elevated D-dimer levels (≥1,000 μg/L), and most of the strokes occurred 8–24 days after the onset of COVID-19 symptoms (25). However, a causal relationship between COVID-19 and ischemic stroke cannot be confirmed, as competing vascular risk factors such as atrial fibrillation were present. Another case series (32) from New York reported on four COVID-19 patients older than 70, all of whom had acute large vessel occlusion. A similar study conducted by Oxley et al. (7) from New York reported five cases of large vessel occlusion in COVID-19. To note, all of these patients are under 50 years of age and only presented with mild symptoms of COVID-19. These findings suggest that COVID-19 primarily causes large vessel occlusion.

## The Potential Mechanism of COVID-19 Related Ischemic Stroke

**Figure 2:**
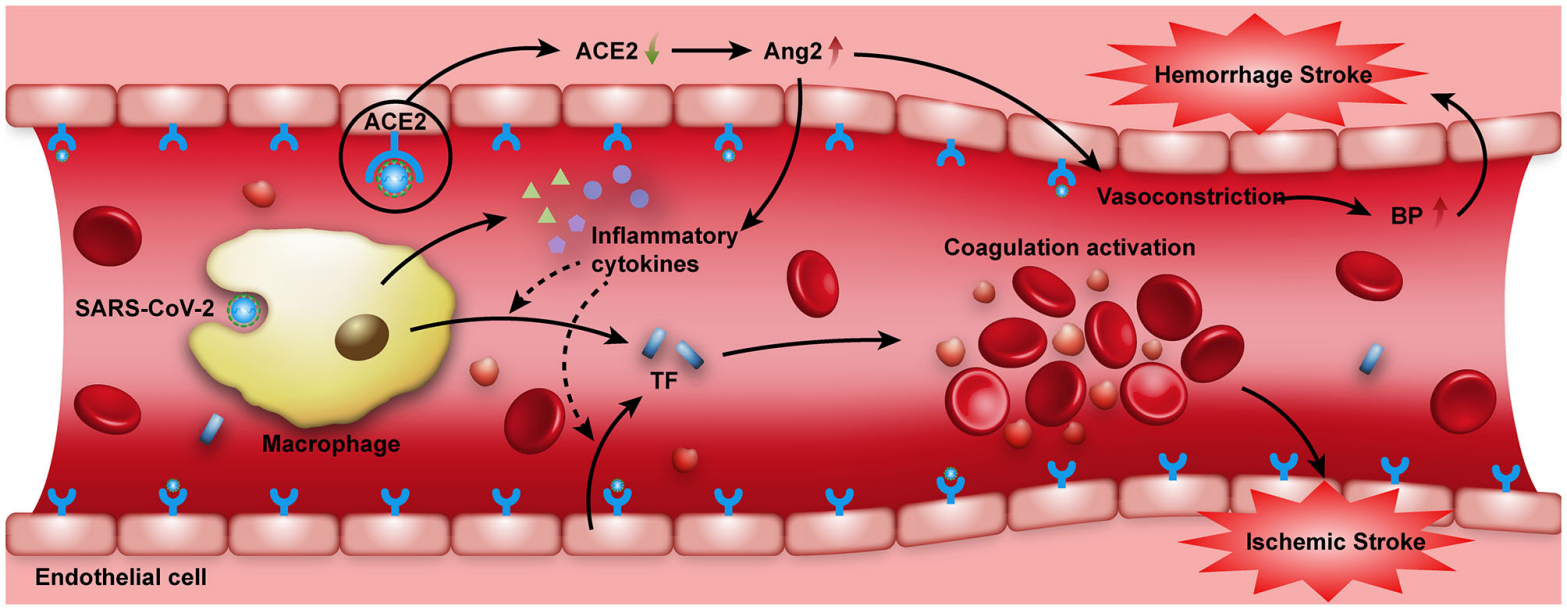
SARS-CoV-2 attacking the vascular system. When the SARS-CoV-2 virus invades the human body, activated monocyte-derived macrophages can release massive amounts of pro-inflammatory cytokines such as IL and TNF to combat the infection. Moreover, when ACE2 receptors on the cell surface are occupied by SARS-CoV-2, the expression and function of ACE2 are reduced, Ang2 in the serum then increases, which also has a pro-inflammatory effect. These pro-inflammatory cytokines can induce the expression of TF. TF expressed by activated monocyte-derived macrophages and endothelial cells can activate the extrinsic coagulation pathway, leading to fibrin deposition and blood clotting. All these factors may increase the risk of acute ischemic stroke. On the other hand, the intracranial cytokine storms induced by SARS-CoV-2 infection may result in the breakdown of the blood-brain-barrier, thus causing hemorrhagic stroke. In addition, the binding of SARS-CoV-2 to ACE2 receptors may increase the synthesis of Ang2, and may thus elevate blood pressure and increase the risk of hemorrhagic stroke. ACE2, angiotensin-converting enzyme II; Ang2, angiotensin II; BP, blood pressure; IL, interleukin; TF, tissue factor; TNF, tumor necrosis factor.

Previous studies indicate that acute bacterial and viral infections, especially respiratory-related infections, are transiently independent risk factors for stroke (33, 34). The association between acute infection and stroke is believed to be caused by the systemic inflammatory response to infection, which can lead to endothelial dysfunction and induce a procoagulant state (34, 35). It has been proposed that the inflammatory response in COVID-19 patients is associated with multiple pathways. As shown in Figure 2, after the infection of SARS-CoV-2, the activated monocyte-derived macrophages can release massive amounts of pro-inflammatory cytokines such as interleukin (IL), tumor necrosis factor (TNF) (35–37). In response to pro-inflammatory cytokines (mainly IL-6), TF is released from monocyte-derived macrophages and endothelial cells (35, 38, 39). TF is known to activate the extrinsic coagulation pathway and leads to fibrin deposition and blood clotting. Moreover, when ACE2 is endocytosed together with SARS-CoV, ACE2 on cells is reduced, followed by an increase of serum angiotensin II (Ang2), which will also induce a pro-inflammatory effect (40–42). The markedly elevated D-dimer levels in patients with acute onset of ischemic stroke also supports that SARS-CoV-2 may cause an acute inflammatory response in the blood vessel walls and trigger a hypercoagulation state.

The traditional causes of stroke in these patients with COVID-19 infection cannot be overlooked. Etiologically, ischemic stroke is caused by cardioembolism, artery-to-artery embolism, or *in-situ* small vessel disease (43). The TOAST classification system has classified ischemic stroke into five subtypes: large artery atherosclerosis, cardioembolism, small artery occlusion, stroke of other determined etiology, and stroke of undetermined etiology (44). Atherosclerosis in patients with a COVID-19 infection may increase the risk of ischemic stroke as a viral infection can potentially destabilize atherosclerotic plaques through systemic inflammatory responses, a cytokine storm, as well as specific changes of immune cell polarization toward more unstable phenotypes (45). In addition, COVID-19 infected patients with cardiovascular comorbidities may have the potential risk of dysrhythmia, which can cause cardioembolism and increase the risk of stroke. A study from Northern Italy showed that the rate of thromboembolic events was higher in COVID-19 patients with a history of cardiovascular disease (23 vs. 6%) (46). What's worse, as COVID-19 is known to have a great effect on the cardiovascular system, the subsequent cardiac dysfunction needs to be considered (13, 47). A recent study reported that 16.7% of 138 hospitalized COVID-19 patients developed dysrhythmia, which presented as a common complication (8). Moreover, viral infections could induce metabolic dysfunction, myocardial inflammation, and activation of the sympathetic nervous system, which would contribute to the development of dysrhythmia (45).

## COVID-19 Infection With the New Onset of Hemorrhagic Stroke

There are fewer cases of hemorrhagic stroke compared with ischemic stroke in patients with COVID-19, and it remains uncertain whether hemorrhagic stroke is directly related to COVID-19 infection.

Sharifi-Razavi et al. (48) reported a case of a 79-years-old man with a fever and cough who developed acute loss of consciousness 3 days later. The patient had a blood pressure of 140/65 mmHg at admission and no history of hypertension or anticoagulation therapy (48). A cerebral CT scan revealed a massive intracerebral hemorrhage (ICH) in the right hemisphere, accompanied by an intraventricular and subarachnoid hemorrhage (48). An oropharyngeal swab confirmed COVID-19 infection, however, a CSF analysis was not performed in this case.

The retrospective study (31) of 11 COVID-19 patients with acute cerebrovascular disease from Wuhan, previously mentioned, also reported on a 60-years-old male who developed cerebral hemorrhage 10 days after severe COVID-19 infection. The patient had increased blood pressure (150/80 mmHg) and died 13 days after the stroke (31).

Poyiadji et al. (49) reported on a female in her late 50's who was diagnosed with COVID-19-associated acute necrotizing hemorrhagic encephalopathy (ANE). Her brain MRI images showed hemorrhagic rim enhancing lesions in the bilateral thalami, medial temporal lobes, and subinsular regions (49). However, testing for the presence of SARS-CoV-2 in the CSF was not performed (49). ANE is a rare CNS complication secondary to viral infections. It has been related to intracranial cytokine storms, which result in blood-brain-barrier breakdown but without direct viral invasion or parainfectious demyelination (49). As evidence shows that severe COVID-19 infection may be associated with cytokine storms (36), we need to be alert to these patients with regard to the occurrence of ANE and other nervous system diseases induced by intracranial cytokine storms.

## The Potential Mechanism of COVID-19 Related Hemorrhagic Stroke

ACE2 is known as a critical enzyme in the renin-angiotensin system (RAS) that regulates blood pressure, fluid, and electrolyte balance, and vascular resistance. It is also the inactivator of Ang2 (50, 51). As shown in Figure 2, the downregulation of ACE2 expression during SARS-CoV-2 infection may increase Ang2 in the serum, which can impair endothelial function and contribute to dysregulation of blood pressure, thus increasing the risk of hemorrhagic stroke (52). As for patients with hypertension, the expression of ACE2 is already low; when SARS-CoV-2 binds to ACE2 receptors, the ability of ACE2 to lower blood pressure is concomitantly reduced, so COVID-19 infection is more likely to induce a cerebral hemorrhage in such patients (53, 54). Thus, it is reasonable to hypothesize that in patients with COVID-19, the cytokine storm and elevated blood pressure can increase the risk of hemorrhagic stroke. However, whether or not hemorrhagic stroke is directly related to COVID-19 infection is difficult to ascertain.

## Management of COVID-19 Infection With Stroke

Neurologists and neurosurgeons worldwide are sharing their experience with the management of COVID-19 patients with neurological manifestation (55–60). What we need to note is that due to the delay in hospital admission caused by the screening of potentially infected patients during the epidemic, stroke patients may miss the optimal therapeutic window.

As ischemic stroke can occur in a systemic prothrombotic state under COVID-19 infection, anticoagulant treatment seems to be reasonable. In the retrospective study from Li et al. (31) previously mentioned, of the 10 patients with ischemic stroke, four received anticoagulant treatment with low-molecular-weight heparin (LMWH) and only one of them died, while six received antiplatelet treatment with Aspirin or Clopidogrel and three of them died. A retrospective analysis of 449 patients with severe COVID-19 performed by Tang et al. (61) revealed that anticoagulant therapy mainly with LMWH appears to be associated with a better prognosis in severe COVID-19 patients meeting sepsis-induced coagulopathy criteria or with markedly elevated D-dimer. The case series (25) from the UK also supports early therapeutic anticoagulation with LMWH. A systematic review of COVID-19 literature reporting on measures of clotting activation also suggests that for COVID-19 patients with elevated D-dimer, antithrombotic treatment may be used (62). However, the efficacy and safety of these anticoagulants in patients with COVID-19 require further investigation, with particular consideration for the risk of bleeding. A detailed assessment of the coagulation profile is necessary. It also needs to be determined by the comprehensive judgment of TOAST classification, clinical syndrome, National Institutes of Health Stroke Scale (NIHSS) score, and laboratory findings.

Thrombectomy also plays a crucial role in treating acute stroke patients. However, endovascular treatments have been reduced in stroke units during the epidemic era. A significant decrease of 61% in the number of patients for thrombectomy was observed in a multicenter study (15). Yaeger et al. reported 10 patients with large vessel occlusion undergoing thrombectomy with a successful reperfusion rate of 90% and concluded that thrombectomy continues to be an effective therapy (63). A study on acute ischemic stroke patients with large artery occlusion who underwent endovascular thrombectomy (EVT) showed that the rate of successful reperfusion was not significantly different in the pre-pandemic group vs. the pandemic group [88.2% (*n* = 30) vs. 85.7% (*n* = 18) (64)]. However, the successful reperfusion rate between COVID-19 infected patients and non-infected patients was unknown. Wang et al. reported on five patients with COVID-19 with large vessel occlusions who underwent EVT and concluded that those patients were more likely to have worse radiographic and clinical outcomes after EVT (64, 65). It cannot be denied that reperfusion therapy during the COVID-19 pandemic could be challenging and that personal protective equipment is necessary to minimize the infection of healthcare workers.

Blood pressure destabilization increases the incidence of heart failure, stroke, and other cardiovascular diseases (66, 67). Accordingly, the management of blood pressure might require specific attention during the hyper-acute and acute stroke phases (68). Angiotensin II receptor blockers (ARBs) and angiotensin-converting enzyme inhibitors (ACEIs) are both antihypertensive drugs for blocking the RAS and lowering blood pressure. Recently, there has been a debate on whether the use of ACEIs/ARBs increases the expression of ACE2, thereby increasing the susceptibility to SARS-CoV-2 (69–71). A single-center retrospective study (72) on the effects of ARBs and ACEIs on COVID-19 patients with pre-existing hypertension showed that ARBs/ACEIs treatment significantly reduced the concentrations of CRP [11.5 (4.0–58.2) vs. 33.9 (5.1–119.2); *P* = 0.049] and procalcitonin [0.061 (0.044–0.131) vs. 0.121 (0.052–0.295); *P* = 0.008], when compared with non-ARBs/ACEIs treatment. Furthermore, a lower proportion of critical patients (9.3 vs. 22.9%; *P* = 0.061), and a lower death rate (4.7 vs. 13.3%; *P* = 0.216) were observed in the ARBs/ACEIs group, although these differences failed to reach statistical significance (72). These findings thus support the use of ARBs/ACEIs in COVID-19 patients with pre-existing hypertension (72). Some literature reviews also support the use of ARBs/ACEIs in COVID-19 patients (73, 74). Other treatments that target the RAS system may also be promising therapies for COVID-19 (73, 75). For example, angiotensin (1–7) has already shown promise in preclinical stroke models and it is in a clinical trial for patients with COVID-19 (NCT04332666). Recombinant human ACE2 (APN01), developed in 2010, has been demonstrated to be able to reduce levels of both Ang2 and IL-6 in a phase II study of acute respiratory distress syndrome. It is also under investigation in China in severe cases of COVID-19 infection (76). On the other hand, treatment of hypertension patients with ACEIs or ARBs can reduce the synthesis or function of Ang2, thus downregulating the production of inflammatory cytokines (77), which may benefit COVID-19 patients with stroke. As there are fewer reports of hemorrhagic stroke in COVID-19 infection, most of the suggestions are concluded from small and retrospective analyses, and more clinical trials are needed to determine the safety and efficacy of these medicines.

To conclude, stroke involves multiple pathophysiological mechanisms. Although COVID-19 may directedly lead to stroke, the common vascular risk factors cannot be overlooked. The management protocol for COVID-19 patients with stroke should also depend on the traditional guidelines. Furthermore, the importance of the use of personal protective equipment and other strategies to minimize exposure during the treatment of stroke patients with COVID-19 cannot be understated.

## Conclusion

Based on current evidence, the causative relationship between cerebrovascular events and COVID-19 is not conclusive. However, previous studies show that acute inflammatory response to COVID-19 infection could induce a procoagulant state and increase the risk of ischemic stroke. Furthermore, the cytokine storm and abnormally elevated blood pressure resulting from the reduction of ACE2 caused by SARS-CoV-2, can increase the risk of hemorrhagic stroke. Given that SARS-CoV-2 could interact with ACE2 expressed in the capillary endothelium, the virus may also damage the blood-brain barrier and enter the CNS. The occurrence of cerebrovascular events is, potentially, related to a direct effect of the viral infection itself. Thus, it is prudent to account for cerebrovascular events and cerebrovascular risk factors as crucial components in the risk model for COVID-19 infection. More studies are needed to establish the mechanisms of cerebrovascular diseases associated with COVID-19. Strategies are urgently needed for specific stroke management during the COVID-19 outbreak and to ensure that stroke patients can get appropriate treatment in time.

## Author Contributions

ZiW and YY designed the study. ZiW, YY, XL, BG, and WL contributed to drafting the article. ML and WL revised the manuscript and polish the language. ZC and ZhW contributed to the critical revision of the article. All authors contributed to the article and approved the submitted version.

## Conflict of Interest

The authors declare that the research was conducted in the absence of any commercial or financial relationships that could be construed as a potential conflict of interest.
